# Negative Aspects of Dietary Habits in Children and Adolescents with Autism Spectrum Disorders

**DOI:** 10.3390/nu16183059

**Published:** 2024-09-11

**Authors:** Martina Grot, Agnieszka Białek-Dratwa, Karolina Krupa-Kotara, Mateusz Grajek, Maciej Nigowski, Elżbieta Szczepańska, Oskar Kowalski

**Affiliations:** 1 Doctoral School, Silesian Medical University in Katowice, 15 Poniatowskiego St., 40-055 Katowice, Poland; 2 Department of Human Nutrition, Department of Dietetics, Faculty of Public Health in Bytom, Silesian Medical University in Katowice, 19 Jordana St., Zabrze-Rokitnica, 41-808 Katowice, Poland; abialek@sum.edu.pl (A.B.-D.); eszczepanska@sum.edu.pl (E.S.); okowalski@sum.edu.pl (O.K.); 3 Department of Epidemiology, Faculty of Public Health in Bytom, Silesian Medical University in Katowice, 41-902 Bytom, Poland; kkrupa@sum.edu.pl; 4 Department of Public Health, Faculty of Public Health in Bytom, Silesian Medical University in Katowice, 41-902 Bytom, Poland; mgrajek@sum.edu.pl; 5 Student Scientific Circle, Department of Public Health, Faculty of Public Health in Bytom, Silesian Medical University in Katowice, 41-902 Katowice, Poland

**Keywords:** diet, autism spectrum, children, food selectivity

## Abstract

Background/Objectives: Diet therapy is a key component of a holistic approach to the physiological and psychological health of children with autism spectrum disorders. A personalized diet, tailored to clinical symptoms, yields positive therapeutic effects. The aim of this study was to assess the intake of specific food groups and the dietary methods used among children and adolescents with autism. Methods: This study included 141 patients from neuropediatric centers diagnosed with autism spectrum disorders. Inclusion criteria were parental consent for the study, age of the child, and autism diagnosis. The research tool was a custom questionnaire covering patients’ demographic data, type of disorder, and a qualitative analysis of the dietary habits of selected products. Results: The predominant dietary models were the basic diet (*n* = 26; 72.22%) and the elimination diet (*n* = 9; 25%), with a higher application rate among children with autism spectrum disorders. Aversion to specific foods/products was more prevalent in children with autism (*n* = 79; 75.24%) compared to those with other neurodevelopmental disorders (*n* = 10; 27.78%). Snacking between meals was significantly more common among the overall neuropediatric patient group (*n* = 140; 99.29%), with fruit purees (*n* = 33; 23.57%) and fruits (*n* = 28; 20%) being the most frequently consumed snacks. Conclusions: The qualitative analysis of dietary habits revealed selective food aversions and eating disorders due to a narrow range of frequently consumed products within dietary groups.

## 1. Introduction

Sensory integration disorders in the oral cavity predispose individuals to disturbances in the proprioceptive system, leading to various perception issues. Sensory integration therapy is essential due to the hypersensitivity and hyposensitivity experienced by this pediatric group. This therapy focuses on two types of dysfunctions related to the registration and modulation of sensory stimuli. A diet rich in ingredients with high antioxidant potential can help to reduce oxidative stress by neutralizing free oxygen radicals, which can ultimately lower blood homocysteine levels and reduce the risk of cardiovascular disease. These ingredients, such as polyphenols, can also stimulate the activity of antioxidant enzymes, which contributes to removing excess free oxygen radicals from the body and further reducing oxidative stress. A diet rich in bioactive ingredients can influence changes in homocysteine and methionine metabolism and increase the activity of antioxidant enzymes, which benefits overall health and reduces the risk of cardiovascular disease [1,2].

In the therapy of neuroatypical children, it is crucial to introduce a diet rich in bioactive components with high antioxidant potential. These can alter the metabolism of homocysteine and methionine and the activity of enzymes such as glutathione peroxidase and superoxide dismutase. These changes may lead to mitochondrial and membrane dysfunctions in neurodevelopmental disorders, particularly autism spectrum disorders [3]. Nutraceuticals are an integral part of treating neurodevelopmental disorders, regulating neuro–hormonal–immunological processes with antioxidants. Important nutrients for treating neuronal–cognitive dysfunctions in children with neurodevelopmental disorders include vitamin B12 (methylcobalamin—MB12 0.9–2.4 μg/d), vitamin B9 (folic acid ≥ 5 mg/d), vitamin B6 (pantothenic acid 0.5–1 mg/d), iron (Fe 7–12 mg/d), zinc (Zn 3–9 mg/d), iodine (I 90–150 μg/d), potassium (K 400–3000 mg/d), magnesium (Mg 30–360 mg/d), vitamins A (800–2000 μg/d), E (100–220 mg/d), D (400–2000 IU/d), C (40–50 mg/d), melatonin (1–3 mg/d), taurine (0.5–2 g/d), carnitine (100 mg/kg body mass), tryptophan (6–8 mg/kg body mass) and glutamine blend (0.8–10 g/kg body mass), and essential fatty acids (omega-3 and omega-6 250–800 mg/d) and cod liver oil [4,5,6].

Scientific reports highlight the importance of specialized nutritional intervention in children with autism. Food and taste selectivity require sensory satiety based on sensory experiences and sensory issues. The sensory diet model includes motor–sensory activities and food training, considering stimulus control, metabolic and allergic factors, and normalizing the nervous system in daily situations [7]. According to the polysensory theory, 85% of disorders in children with autism involve the sense of touch and proprioception, while 25% involve the senses of smell and taste. Regulating sensory satiety should minimize behavioral–cognitive habits and diversify food without distracting stimuli, avoiding monotony and negatively perceived products [8,9]. Current clinical reports in the field of complementary and alternative medicine do not present a nutritional intervention in the form of a sensory diet individualized on the basis of an analysis of biochemical parameters in pediatric patients. The subsequent consequences lead to the implementation of inadequate diet therapy based on a deficient, elimination model of nutrition with the occurrence of food selectivity. Therefore, there is a need to expand and create scientific research on the feeding patterns of children with neurodevelopmental spectrum disorders in the discipline of health sciences with a pediatric specialization. In cases of food selectivity, the sensory diet model uses sensory play with aversive products, such as vegetables, for a minimum of 2 weeks, activating the senses of hearing and sight through storytelling. Visualization of disliked products stimulates the sensory signal transmission process. Sensory stimulation through taste, touch, smell, and hearing increases food intake, e.g., a mix of dark green vegetables with fruits creating a sweet taste [10,11,12,13,14].

Biomarkers obtained from alternative and elimination diets raise controversy regarding their therapeutic potential in autism (ASD). Research emphasizes the need for further evaluation of ketogenic diets, which eliminate casein and gluten (GFCF) or just casein or gluten. Combining a gluten-free diet with a modified ketogenic diet may offer therapeutic effects in some ASD patient subgroups [15]. The aim of this study was to assess the intake of specific food groups and the dietary methods used among children and adolescents with autism.

## 2. Materials and Methods

### 2.1. Study Group

The cross-sectional study was conducted between October 2021 and April 2022. Inclusion criteria were parental or legal guardian consent, the child’s age up to 17 years, and a diagnosis of autism spectrum disorder and other neurodevelopmental disorders. Exclusion criteria included the absence of consent, under 17 years of age, and lack of a neurodevelopmental diagnosis. Based on these criteria, 141 children were included in the final analysis. Despite standardized ICD-11 criteria for autism spectrum diagnosis, patients were classified into two groups: autism spectrum disorder (ASD) (*n* = 105) and other neurodevelopmental disorders including Asperger’s syndrome, Rett syndrome, selective mutism, and attention-deficit/hyperactivity disorder (ADHD) (*n* = 36). The pediatric age range was from 1 to 17 years. Age distribution was categorized into 5-year groups: 0–3 years (*n* = 35; 24.82%), 3–7 years (*n* = 62; 43.97%), 7–11 years (*n* = 27; 19.15%), 11–15 years (*n* = 13; 9.22%), and 15–17 years (*n* = 4; 2.84%). Children from the postnatal period to 17 years of age with neurodevelopmental disorders were included. This study is a continuation of a research project on feeding and nutrition disorders in children and adolescents with autism spectrum disorders. The sample size was calculated according to the formula Nmin = NP·(α2·f(1 − f)) ÷ NP·e2 + α2·f(1 − f), where Nmin—minimum sample size; NP—the size of the population from which the sample is drawn; α—confidence level for the results; f—the size of the fraction; e—assumed maximum error. The population of Poland is ≈38 million, people and epidemiological data show that the incidence of neurodevelopmental disorders is 5 per 10,000 people. According to calculations, the population of people with neurodevelopmental disorders is ≈19,000 people. The minimum sample size of respondents was calculated, which was 138 (α = 0.95; f = 0.9; e = 0.05). Based on these calculations, the collected group was considered representative. Normality was assessed by the absence of differences between subgroups (*p* > 0.05).

### 2.2. Study Location

The study locations were randomly selected. Cluster sampling was used, based on the specialization of the medical facilities in the pediatric group. Out of 55 institutions specializing in behavioral–cognitive disorders with a focus on pediatric neurology in the Silesian Agglomeration in Poland, three facilities were chosen: the Upper Silesian Children’s Health Center in Katowice, the Municipal Hospital Group in Chorzów, and the Statera Physiotherapy Center in Katowice.

### 2.3. Sample Selection

The sample selection followed ethical principles for research involving human participants in accordance with the Helsinki Declaration. The study was conducted using a questionnaire and included retrospective data. Additionally, consent was obtained from the Bioethics Committee of the Medical University of Silesia in Katowice for the publication of the results. The Bioethics Committee approved the study titled “Feeding Disorders and Oral Sensitivity in the Context of Sensory Feeding in Patients with Neurodevelopmental Disorders” (Resolution No. PCN/CBN/0052/KB1/142/22/23).

### 2.4. Research Tool

The research tool was a questionnaire developed by the author, completed individually by a clinical pediatric dietitian through a medical-nutritional interview with the parent of each pediatric patient suffering from neurology. The questionnaire consisted of 53 questions and included the following items: demographic data, clinical data (diagnosis of the disorder), course and management, food intolerances and allergies, dietary model with product consumption frequency, and hydration level. The nutritional interview assessed qualitative dietary analysis, divided into 12 food groups according to the author’s classification with the 13th group, which includes water and drinks (I—cereals, II—milk and dairy products, III—eggs, IV—meat, fish, cold cuts, and offal, V—butter and cream, VI—other fats—rapeseed oil, sunflower oil, linseed oil, sesame oil, evening primrose oil, thistle oil, pumpkin seed oil, avocado oil, olive oil, soft margarine, mayonnaise and dressings, i.e., salad dressings, VII—potatoes, VIII—vegetables categorized into cooked and raw and vegetables with low glycemic index—cabbage, brussels sprouts, cauliflower, broccoli, tomato, pepper, horseradish, raw carrot, spinach, sorrel, green lettuce, green beans, green peas, garlic, chives, chicory, dill, parsley, kale, onion, leek, root parsley, celery (raw), cucumber, radish, zucchini, squash, rhubarb; medium glycemic index—sweet potatoes, beetroot (cooked), corn; high glycemic index—potatoes, kohlrabi, carrot (cooked), pumpkin, celery (cooked), IX—fruits categorized into fruits with low glycemic index—chokeberry, currant, gooseberry, strawberry, wild strawberry, raspberry, blackberry, orange, grapefruit, tangerine, cranberry, rose hip, peach, apple, blueberry, pear, plum, cherry, sour cherry, unripe bananas; medium glycemic index—kiwi, apricot, pineapple, grape, ripe banana, melon; high glycemic index—watermelon, and dried fruits—figs, dates, apricots, plums, raisins, X—legumes, XI—seeds, nuts, and grains, XII—sugar, sweets, snacks), using a six-point frequency scale: “never”, “once a month”, “a few times a month”, “a few times a week”, “daily”, “a few times a day”. The child’s feeding pattern was also assessed by asking the parent about the type of diet.

### 2.5. Statistical Analysis

The nutritional interview questionnaire was validated. In order to validate the questionnaire and to check the relevance and acceptability of the questions contained in it, a pilot study was conducted on a group of 15 mothers and their children. The reproducibility of the answers was checked by comparing the answers in the same group of subjects. The repeat pilot study took place one month after the pilot study to avoid freshness effects. To assess the reproducibility of the results, the x-parameter (Kappa) was calculated (results from the pilot study and the repeat pilot study). A very good (x ≥ 0.80) or good (0.79 ≥ x ≥ 0.60) response agreement was obtained for most questions. For the seven questionnaire questions analyzed, the concordance between the results obtained at the beginning of the study and in the re-test was low (x < 0.2), and these questions were removed from the questionnaire. The Cronbach’s α coefficient for the standardization sample was also analyzed and was 0.79, indicating a high reliability of the questionnaire.

Data from the medical-nutritional interviews were entered into a database created in Microsoft Excel. Descriptive statistics were then applied. Statistical analysis was conducted using STATISTICA 13.0 software by Stat Soft, Krakow, Poland. Qualitative data were characterized using statistical dependencies with Chi^2 NW, Chi^2 Pearson tests. The strength of relationships was determined using Cramer’s V, Phi-Yule, and Gamma coefficients. Statistical significance of differences was assessed using *p*-values, with significance defined at *p* < 0.05.

## 3. Results

### 3.1. Dietary Model

The predominant dietary model was the basic diet (*n* = 69; 65.71%) and the elimination diets including gluten-free, casein-free, and sugar-free (*n* = 28; 26.67%) among children with autism spectrum disorder (ASD). A variety of dietary approaches were observed, including ketogenic diet (*n* = 3; 2.86%), high-fat diet (*n* = 1; 0.95%), low-sodium with appropriate calcium intake (*n* = 1; 0.95%), low-fat diet (*n* = 1; 0.95%), high-protein diet (*n* = 1; 0.95%), and vegetarian diet (*n* = 1; 0.95%). Among children with other neurodevelopmental disorders (Asperger’s syndrome, Rett syndrome, ADHD, selective mutism), the basic diet (*n* = 26; 72.22%) and elimination diet (gluten-free, casein-free, sugar-free) (*n* = 9; 25%) were also most prevalent. The least frequent dietary approach was vegetarian diet (*n* = 1; 2.78%).

### 3.2. Phytotherapeutic Process

Autistic patients did not use bioactive compounds such as phytotherapeutics, herbs, and infusions (*n* = 92; 87.62%). However, a small percentage of children used phytotherapeutics (*n* = 13; 12.38%) including chamomile, lemon balm, sage, mint, white mulberry, Ginkgo biloba, Cistus, nettle, field horsetail, tansy, nanoparasite, ashwagandha, woad, and African geranium, pau d’arco, and oregano. Similarly, a larger proportion of children with other neurodevelopmental disorders also did not use bioactive compounds in the form of phytotherapeutics, herbs, and infusions (*n* = 30; 83.33%). However, a small percentage used phytotherapeutics (*n* = 6; 16.67%) including chamomile, nettle, linden, lemon balm, mint, and black walnut extract.

### 3.3. Qualitative Analysis of Dietary Intake of Selected Food Groups

The consumption level of products from Group I (cereals) was predominantly daily (*n* = 45; 42.86%) for refined bread, and fine-grain groats were consumed a few times a week (*n* = 38; 36.19%) in the autism spectrum group. In the pediatric group with other neurodevelopmental disorders, refined bread was also consumed daily (*n* = 15; 41.67%), with wholemeal bread (*n* = 13; 36.11%) and fine-grain groats (*n* = 15; 41.67%) consumed a few times a week (Table 1).

The consumption level of products from Group II (milk and dairy products) was mostly none for all products in this group among children with autism spectrum disorder. In the pediatric group with other neurodevelopmental disorders, the highest consumption level was observed for yellow cheese and processed cheese, which was consumed a few times a week (*n* = 11; 30.56%), and ready-made dairy drinks (*n* = 11; 30.56%), which was consumed a few times a month (Table 2).

The consumption level of products from Group IV (meat, fish, and meat products) was mostly a few times a week for poultry meat (*n* = 43; 40.95%) and pork meat (*n* = 45; 42.86%) and a few times a month for lean fish (*n* = 40; 38.10%) among children with autism spectrum disorder. In the pediatric group with other neurodevelopmental disorders, poultry meat was consumed a few times a week (*n* = 20; 55.56%), pork meat was consumed a few times a month (*n* = 17; 47.22%), and fatty fish was consumed once a month (*n* = 12; 33.33%) (Table 3).

The consumption level of products from Group VIII (vegetables) was mostly a few times a week for cooked vegetables (*n* = 26; 24.76%) and vegetables with a high glycemic index (*n* = 40; 38.10%) and low glycemic index (*n* = 27; 25.71%) were consumed a few times a month in the autism spectrum group. In the pediatric group with other neurodevelopmental disorders, raw vegetables were consumed daily (*n* = 10; 27.78%) and cooked vegetables were consumed a few times a week (*n* = 10; 27.78%) (Table 4).

The consumption level of products from Group IX (fruits) was mostly a few times a week for fruits with a low glycemic index (*n* = 30; 28.57%) and medium glycemic index (*n* = 32; 30.48%) among children with autism spectrum disorder. In the pediatric group with other neurodevelopmental disorders, fruits with both low (*n* = 10; 27.78%) and medium glycemic index (*n* = 13; 36.11%) were also consumed a few times a month (Table 5).

## 4. Discussion

Diet plays a crucial role in neurogenesis and the development of the nervous system in the fetus and child. The formation of the central nervous system depends on the intake of bioactive nutrients such as polyunsaturated fatty acids (linoleic, alpha-linolenic, docosahexaenoic, arachidonic), sphingolipids, phospholipids, iron, zinc, iodine, vitamins A, B12, D, complete proteins, and amino acids (tyrosine, tryptophan, phenylalanine), tailored to the metabolic and clinical needs of pediatric neurology patients [16,17,18,19].

Studies show that neuroatypical individuals exhibit different dietary patterns compared to neurotypical groups, with selective tolerance and sensory sensitivity. Esteban-Figuerola et al. reported lower consumption of proteins, dairy, whole grains, and fatty fish, but higher consumption of fruits and vegetables in children with ASD. Calcium, phosphorus, selenium, and vitamins D, B1, B2, and B12 levels were low, while vitamin E levels were adequate [20]. Lange et al. confirmed limited dietary preferences and more frequent food refusals in children with ASD, with low fruit and vegetable diversity due to disruptions in taste and texture perception [21]. Susan et al. found that children with ASD consume primarily refined products, cereals, and chicken nuggets at a rate of 92% [22].

In addition, the authors of another study by Mari-Bauset et al. presented a limited intake of products from 12 groups along with the most frequent consumption of 11 types of products over a three-day period, such as wheat bread, butter, cream cheese, chicken meat, and fruit with a low glycemic index (apple, cooked vegetables potato, carrot, white rice, butter croissant, wheat roll) [23,24,25]. By verifying our own results, it is possible to conclude that there is similarity in the dietary analysis when comparing the author’s study together with the work of other researchers.

In the study by Lange et al., the implementation of an elimination diet in children with autism reduced gastrointestinal symptoms such as diarrhea, abdominal pain, and bloating. The elimination diet increased the intake of fiber, magnesium, copper, iron, carotenoids, folic acid and vitamin C but decreased the intake of calcium, riboflavin, short-chain fatty acids, and monounsaturated fats, which are harmful to the nervous system [21]. Brzóska et al. and Kazek et al. observed sensory hypersensitivity in 63.4% of children with autism, preferring sweet flavors and uniform meal textures. Other studies have also reported limited consumption of products from 12 groups [23,24]. These results are in line with other studies and our own findings on the diet of children with autism.

On the other hand, the work by Thorsteinsdottir et al. divided their study into a group with and without a neural disorder diagnosis, presenting about 50% in the study group with high unacceptability and selectivity in meal intake together with family conditioning in the form of restricted intake of products also by the parents of the children studied. The level of intake of, among others, green leafy vegetables in the form of kale, salads, spinach, berries, fresh fruit, and nuts was a low level compared to the control group, just as in our own study conducted, in the study group, the intake of spinach and lettuce was a low level with a frequency of several times a month or not at all [26]. Subsequently, an analysis of atypical eating behavior in a study by Susan et al. and Mari-Bauset et al. reported restricted food preferences in 88% of children on the autism spectrum, along with a 25% degree of having three or more non-specific relationships with food [2,25].

In relation to the above study, the self-analysis undertaken shows a parallel to the high levels of food selectivity found by parents of ASD patients (*n* = 79; 75.24%), while other atypical disorders showed slightly lower levels of recognition of aversion to specific foods and fear of new foods (*n* = 26; 72.22%).

The interval between meals was 2–3 h, with frequent snacking (98.59%). Qualitative dietary assessment revealed selectivity in the consumption of whole-grain bread, groats, legume flours, milk, dairy, eggs, fatty fish (halibut, tuna), cold cuts, offal, margarine, nuts, seeds (almonds, pistachios, cashews, pumpkin, sesame, sunflower), boiled potatoes, raw vegetables, and high-glycemic fruits. Higher consumption levels were noted for lean fish (cod, pollock, pike-perch), poultry meat, butter, plant oils, mashed potatoes, cooked vegetables, fruits with low and medium glycemic indices, honey, biscuits, and cakes.

Hydration assessment indicated adequate juice and nectar intake a few times a month but insufficient consumption of medium-mineral, non-carbonated mineral water (once daily). In children with Asperger’s syndrome, selective mutism, Rett syndrome, and ADHD, lower food selectivity was observed compared to those with an ASD diagnosis. Higher intake in children with lower selectivity included whole-grain bread, groats, poultry meat, fatty fish, butter, plant oils, cooked and baked potatoes, raw and cooked vegetables, low-glycemic fruits, and highly processed sweets.

It is essential to implement educational programs on nutrition for parents, creating a varied dietary model and considering nutritional status in the development of neurodevelopmental disorders. Sensory exposure, reintroducing products after a 2–3 day interval, and mixing new products with previously introduced ones are particularly noteworthy [13,27,28]. Education on the health impacts of dietary habits is crucial in the pediatric neurology group.

It is estimated that between 46% and 89% of children with ASD experience diet-related problems involving atypical eating habits, rituals, and food selectivity. Such behaviors often result in a preference for certain foods, which can significantly disrupt the balance of the diet [29,30]. Often, children with ASD show atypical eating behaviors in childhood and follow a more restricted diet than children without ASD [29,31]. Dietary selectivity is, therefore, frequently reported as a common cause of problems and conflicts at mealtimes [29]. Consequently, children with ASD often prefer energy-dense foods, mainly sweetened beverages, while limiting their intake of nutrient-rich foods such as lean protein, fruit, vegetables and fiber-rich foods [30]. The results of the Bandini L. study indicate that children with ASD rejected an average of 47.2% of the foods on the food questionnaire (FFQ), including 62.3% of vegetables and 55.6% of fruit. They consumed an average of 2.2 portions of fruit and vegetables daily, and their dietary repertoire included an average of 19.0 unique foods. These data suggest considerable dietary selectivity, limited dietary diversity, and low fruit and vegetable intake in this group [30]. Research by Ahumad D. also confirms inappropriate eating habits in children with ASD. The diets of these children tend to be limited and unbalanced. Although most consume dairy and cereal daily, vegetables and fruit are much less present in their diet, with 36% of children not consuming vegetables at all. Meat consumption is uneven, with a marked avoidance of fish and eggs. Ultra-processed products and sweets are often present in their diet, while regular water intake is low [29].

The food selectivity associated with ASD creates additional difficulties for caretakers of children with the disorder. Parents note that various aspects of food, such as texture, appearance, brand, packaging, temperature, method of serving, color, taste, and smell, significantly impact their children’s food preferences [31]. Traits include sensory sensitivity, difficulties establishing and maintaining social relationships, problems with self-esteem and identity, difficulties managing emotions, a specific way of thinking typical of autism, and a need for control and predictability. These traits can interact to influence both directly and indirectly a variety of restrictive eating behaviors [32]. In a study by Mayers et al. [22], it was presented that atypical eating behaviors such as restricted food preferences, texture sensitivity, and brand-specific preferences were five times more frequent in autistic adolescents than in children with other disorders (including ADHD intellectual disability, language impairment, and learning disabilities) and 15 times more frequent in the autistic group than in their typically developing peers, with restricted food preferences being the most common atypical eating behavior. Autistic adolescents showed significantly higher rates of feeding problems compared to typically developing and atypical groups (i.e., those with developmental disabilities other than autism), particularly for food selectivity [33]. Most studies comparing children and adolescents with autism with groups with other disorders and/or typical development found that autistic individuals had higher rates of dietary selectivity. In studies without control groups, dietary selectivity rates in samples of autistic adolescents were high, from 57 to 72% [34,35].

This study confirms that children with neurodevelopmental disorders, particularly autism, exhibit specific dietary patterns that can affect their health and neurodevelopment. Dietary preferences in this group are often restricted, leading to deficiencies in some essential nutrients. Dietary models, including elimination diets, are commonly used, although their effects can be positive and negative, requiring further research and individualized therapeutic approaches.

## 5. Conclusions

Qualitative dietary assessment revealed moderate to severe food selectivity and low hydration levels, with a narrow range of consumed products from 12 groups. The basic dietary model predominated, and the eating habits of children with autism and other neurodevelopmental disorders are atypical and differ in sensory profile.

## 6. Strengths and Limitations of the Study

The strengths of this study include the collection of data from a group of patients with neurodevelopmental disorders and individualized medical-nutritional interviews. This study is distinguished by its originality due to the detailed analysis of the diet of patients with autism and other neurodevelopmental disorders. Limitations include the absence of a control group of healthy children and the division of children into age subgroups rather than analysis by age criterion. Nevertheless, considering the diverse age range allows for the identification of negative dietary aspects.

## Figures and Tables

**Table 1:** Level of consumption of products from food group I.

Cereal Products	Type of Disorder		*p*-Value
	Autism Spectrum (*n* = 105)	Other Neurodevelopmental Disorders (*n* = 36)	
Refined bread, so called, light, such as light wheat bread or rye, wheat-rye, toasted bread, plain rolls, rolls and butter croissants	daily—45 (42.86%)	daily—15 (41.67%)	*p* < 0.05—statistical significance
Wholemeal bread or bread with grains, so-called dark bread, e.g., wholemeal rye, graham or rye bread with grains, pumpernickel, grahams, crisp bread	at all—45 (42.86%)	several times a week—13 (36.11%)	
Almond flour, flour from lentils, chickpeas, tapioca	at all—72 (68.57%)	at all—28 (77.78%)	
Fine-grained groats Refined, such as manna, broken barley, pasta, white rice, rice flakes	several times a week—38 (36.19%)	several times a week—15 (41.67%)	
Coarse-grained groats unrefined, such as buckwheat groats, pearl barley, brown rice, wholemeal pasta	at all—37 (35.24%)	several times a month—13 (36.11%)	

*p* < 0.05—statistical significance.

**Table 2:** Level of consumption of products from food group II.

Milk and Dairy Products	Type of Disorder		*p*-Value
	Autism Spectrum (*n* = 105)	Other Neurodevelopmental Disorders (*n* = 36)	
Sweetened milk	at all—102 (97.14%)	at all—31 (86.11%)	*p* > 0.05—NS (no statistical significance)
Sour milk (1.5% fat)	at all—93 (88.57%)	at all—29 (80.56%)	
Sour milk (2% fat)	at all—75 (71.43%)	at all—28 (77.78%)	
Sour milk (3.2% fat)	at all—64 (60.95%)	at all—25 (69.44%)	
Kefir	at all—87 (82.86%)	at all—30 (83.33%)	
Natural yogurt	at all—53 (50.48%)	at all—19 (52.78%)	
Fruit yogurt (self-prepared)	at all—55 (52.38%)	at all—21 (58.33%)	
Ready-made, dairy drinks Sweetened, such as fruit yogurts, yogurts with cereal	at all—54 (51.43%)	at all—14 (38.89%) several times a month—11 (30.56%)	
Half-cottage cheese	at all—65 (61.90%)	at all—22 (61.11%)	
Fat cottage cheese	at all—91 (86.67%)	at all—29 (80.56%)	
Yellow cheese, melted	at all—49 (46.67%)	at all—12 (33.33%) several times a week—11 (30.56%)	

*p* > 0.05—NS (no statistical significance).

**Table 3:** Level of consumption of products from food group IV.

Meat Products (Meat, Cold Cuts, Poultry, Fish)	Type of Disorder		*p*-Value
	Autism Spectrum (*n* = 105)	Other Neurodevelopmental Disorders (*n* = 36)	
Poultry meat (turkey, chicken)	several times a week—43 (40.95%)	several times a week—20 (55.56%)	*p* > 0.05—NS (no statistical significance)
Pork meat	several times a month—45 (42.86%)	several times a month—17 (47.22%)	
Beef meat	at all—66 (62.86%)	at all—17 (47.22%)	
Veal meat	at all—73 (69.52%)	at all—27 (75%)	
Oily fish: salmon, halibut, tuna, mackerel, herring, eel, sprat, sardine	at all—59 (56.19%)	once a month—12 (33.33%) at all—11 (30.56%)	
Lean fish: tench, sole, cod, hake, pikeperch, pollock, mirin, panga, bream, cod	several times a month—40 (38.10%) at all—37 (35.24%)	at all—14 (38.89%) once a month—9 (25%) several times a month—9 (25%)	
Offal	at all—95 (90.48%)	at all—−31 (86.11%)	
Smoked sausages	at all—69 (65.71%)	at all—27 (75%)	
Baked sausages	at all—43 (40.95%)	at all—15 (41.67%)	
Cooked sausages	at all—49 (46.67%)	at all—11 (30.56%) several times a week—9 (25%)	

**Table 4:** Level of consumption of products from food group VIII.

Vegetables	Type of Disorder		*p*-Value
	Autism Spectrum (*n* = 105)	Other Neurodevelopmental Disorders (*n* = 36)	
Cooked vegetables	several times a week—26 (24.76%) daily—26 (24.76%)	several times a week—10 (27.78%) several times a month—9 (25%)	*p* < 0.05—statistical significance
Raw vegetables	at all—37 (35.24%)	daily—10 (27.78%) several times a month—8 (22.22%)	
Vegetables with a low glycemic index glycemic index	several times a month—27 (25.71%) several times a week—25 (23.81%)	several times a month—10 (27.78%) daily—9 (25%)	
Vegetables with a medium glycemic index glycemic index	at all—34 (32.38%) several times a month—31 (29.52%)	at all—12 (33.33%) several times a month—11 (30.56%)	
Vegetables with a high glycemic index	several times a week—40 (38.10%)	several times a month—13 (36.11%)	

**Table 5:** Level of consumption of products from food group IX.

Fruits	Type of Disorder		*p*-Value
	Autism Spectrum (*n* = 105)	Other Neurodevelopmental Disorders (*n* = 36)	
Fruits with a low glycemic index	several times a week—30 (28.57%) daily—29 (27.62%)	several times a month—10 (27.78%)	*p* < 0.05—statistical significance
Fruits with a medium glycemic index	several times a week—32 (30.48%) several times a month—28 (26.67%)	several times a month—13 (36.11%)	
Fruits with a high glycemic index	at all—46 (43.81%)	once a month—15 (41.67%)	
Dried fruit	at all—69 (65.71%)	at all—23 (63.89%)	

## Data Availability

The data presented in this study are available on request from the corresponding author due to privacy.
